# Antibacterial, anti‐inflammatory, analgesic, and hemostatic activities of *Acanthopanax trifoliatus* (L.) merr

**DOI:** 10.1002/fsn3.2190

**Published:** 2021-02-28

**Authors:** Zefeng Chen, Shupeng Cheng, Huiqiong Lin, Wenjing Wu, Liyi Liang, Xicai Chen, Xi Zheng, Yan He, Kun Zhang

**Affiliations:** ^1^ Allan H. Conney Laboratory for Anticancer Research School of Biomedical and Pharmaceutical Sciences Guangdong University of Technology Guangzhou China; ^2^ Susan Lehman Cullman Laboratory for Cancer Research Department of Chemical Biology Ernest Mario School of Pharmacy, Rutgers The State University of New Jersey Piscataway NJ USA; ^3^ School of Biotechnology and Health Sciences Wuyi University Jiangmen China

**Keywords:** *Acanthopanax trifoliatus* (L.) Merr, analgesic, antibacterial, anti‐inflammatory, hemostatic

## Abstract

*Acanthopanax trifoliatus* (L.) Merr (AT) is a medicinal and edible plant with high nutritional value. The biological activity of *A. trifoliatus* (L.) Merr and its basis for injury treatment are explored in this paper. AT was ethanol‐extracted then refined separately with petroleum ether, chloroform, ethyl acetate, and n‐butanol. Active ingredients were analyzed, and anti‐bacterial, anti‐inflammatory, analgesic, and hemostatic effects were explored. Petroleum ether layer (PEL) extract produced the strongest antibacterial effect. Ethyl acetate layer (EAL) extract had the highest active substance content, with strong hemostatic and analgesic activities. Chloroform layer (CL) extract had the strongest anti‐inflammatory effect and significantly reduced IL‐1β protein expression. Active ingredients were analyzed using HPLC and UPLC‐MS to determine saponin, polyphenol, flavonoid, and characteristic ingredient contents. EAL extract had the highest polyphenol and flavonoid levels, including rutin, chlorogenic acid, isochlorogenic acid A, and isochlorogenic acid C, which may contribute to its nutritional activities. The study provides a reliable theoretical and practical basis for the applications of AT nutraceutical products.

## Graphical abstract

Organic solvent layer extracts of *Acanthopanax trifoliatus* (L.) Merr (AT) have various biological activities. Extracts produced strong antibacterial, anti‐inflammatory, analgesic, hemostatic activities and reduced IL‐1β protein expression. These results provide a reliable theoretical and practical basis for the research and development of a topical application of AT products.

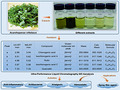

## 1 INTRODUCTION

*Acanthopanax trifoliatus* (L.) Merr (AT) belongs to *Acanthopanaceae* family, which is widely distributed in Asian countries such as Thailand, Malaysia, Vietnam; it is also found in the South, Southwest, and the middle of China (Hamid, Kee, & Othman, 2013). AT is an edible plant with high nutritional value and has been long used as a fresh vegetable and as a medicinal material (Wang et al., 2015). AT has over 15 inorganic elements with a high content of potassium and calcium, low level of sodium, 2–3 times more vitamin C, and 8 times higher Fe content than tomato (Gao et al., 2018). It also contains over 100 compounds of which polyphenols and saponins are the main active components, as well as volatile oil, polysaccharides, proteins, crude fibers, reducing sugars, and others. The benefit of polyphenols, especially flavonoids, on human health has been paid more and more attention. They are the important material basis of pharmacological activities such as anti‐oxidation, anti‐inflammatory, cell protection, anti‐hyperglycemia, anti‐hyperlipidemia, and anti‐cancer (Sithisarn et al., 2016; Hamid et al., 2013).

Flavonoids have bacteriostatic effects due to their weak alkalinity, which coagulates or deforms proteins (Tian et al., 2018). Wang Yan found that the antimicrobial effect of flavonoids derived from alcohol‐elution methods was stronger than that from water, and that the polysaccharide extract showed no obvious antimicrobial effects (Sithisarn et al., 2016). The total flavonoids from *Acanthopanax vulgaris* had the strongest bacteriostatic effect on *Escherichia coli*, followed by *Staphylococcus aureus* (Jie et al., 2016). The extracts from different parts of Thai *Acanthopanax* have strong in vitro antioxidant activity, and the root bark and tender leaves display the strongest free‐radical scavenging activity (Sithisarn et al., 2011). Thai *Acanthopanax* displayed stronger DPPH free‐radical scavenging activity than *Acanthopanax senticosus* and *Acanthopanax trifoliata* from Taiwan (Lee et al., 2004). Purified flavonoid liquor from *Acanthopanax vulgaris* at 45 mg/ml had significantly greater antioxidant effects than that of vitamin C (Maolian et al., 2002). Hamid RA’s team in Malaysia observed the inhibition rate of AT ethanol extract was 46.23% in a carrageenan‐induced rat swelling inflammation model, and 45.7% in a CFA‐treated rat long‐term inflammation model; this effect was comparable to the anti‐inflammatory effects of piroxicam and indomethacin (Hamid et al., 2013). Furthermore, Yang Huiwen found that 10, 20, and 40 mg/(kg·d) of oral flavonoid extract from AT could significantly inhibit carrageenan‐induced foot swelling in rats in a dose‐dependent manner (Yang et al., 2014). Our team found that AT extracts had anticancer activities in prostate cancer cells, neural cancer cells, breast cancer cells, liver cancer cells, and large cell lung cancer cells. We also provided evidence that AT terpenoids exert anticancer activities, indicating that AT may be a useful edible plant for further development as a health supplement (Li et al., 2016).

AT is widely used in folk medicine in Asia for bruises, tuberculosis, ulcer healing, tinea, and physical fitness. In China, AT is commonly used for soup and can be further processed into teas (Nan, Koen, Gabriele, & Claus, 2012). In Thailand, AT is commonly known as “Phak Paem” and is used as a traditional vegetable and folk herb (Sithisarn et al., 2011). In Vietnam, AT has traditionally been used for its positive therapeutic effects, particularly as an anti‐inflammatory agent (Quan et al., 2003). AT is a raw material used in winemaking and as a hot beverage in Taiwanese folk tradition (Chien et al., 2015). It is also one of the components of traditional Malaysian Hakka tea, *lei cha*, due to its medicinal benefits for the common cold, jaundice, stomachache, and diarrhea. In Cambodia, Laos, and Vietnam, herbal extracts from AT bark are used to alleviate anxiety and improve memory (Muselli et al., 2015). AT has been fortified into the soaps to relieve itching and fluoride toothpaste for periodontitis treatment (Huang, 2007). It can also be used for neonatal eczema, with therapeutic effects as an antipyretic, anti‐inflammatory, and detoxification aid (Min et al., 2019).

AT has been used to treat bruises, neuralgia, impotence, and gout for a long time. However, there are few pharmacological studies on the biological activities of AT. In this study, the main active components, and the antibacterial, anti‐inflammatory, analgesic, and hemostatic activities of Southern Chinese AT extracts are investigated. This research provides a reliable theoretical and practical method for studying AT, which will be of great significance for the development of functional AT products.

## 2 MATERIALS AND METHODS

### 2.1 Materials

Standard gallic acid, rutin, and ciprofloxacin were purchased from Sigma‐Aldrich Co.. 12‐O‐tetradecanoylphorbol‐13‐acetate (TPA) was obtained from Shanghai Beyotime Biotechnology Co., LTD. Diclofenac gel (1%) was purchased from Guangdong Dashenlin Pharmaceutical Co., LTD. All other chemicals used were reagent grade.

ATs are collected from different areas in the south of China, such as Enping, Yangjiang, and Shantou in Guangdong Province, Xishuangbanna in Yunnan Province, Shaowu and Longyan in Fujian Province). The six batches of ATs from Enping (20,160,315, 20,160,401, 20,160,429, 20,160,504, 20,160,601, and 20,160,618) were picked in production periods from mid‐March, early April, late April, early May, early June, and mid‐June, respectively.

### 2.2 Preparation of extracts

**FIGURE 1:**
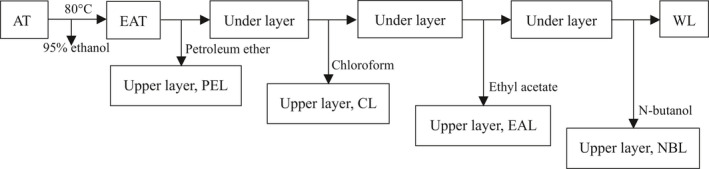
The extraction process used for EAT, PEL, CL, EAL, NBL, and WL

Dried AT stems and leaves (600 g) were boiled in 95% ethanol (w/v) for 2 hr and then filtered to collect the ethanol extract (EAT). The crude ethanol extract was further extracted with organic solvents with different polarities, in turn, to collect the petroleum ether layer (PEL), chloroform layer (CL), ethyl acetate layer (EAL), n‐butanol layer (NBL), and water layer (WL), respectively (Figure 1). The obtained extracts were dried at 40℃ in the vacuum, weighed, and the yield was calculated.

### 2.3 Determination of saponin, polyphenol, and flavonoid content

Dry AT or extracts (equivalent to 50 mg/ml of AT) were incubated in 35% ethanol at 80℃ for 2 hr and then sonicated for 5 min. The sample solution was dissolved completely in 35% ethanol solution and diluted after filtration for further investigation. Ultraviolet spectrophotometry (Lambda 25, Perkin Elmer) was used to determine the total saponin, polyphenol, and flavonoid content. These methods were also used for content determination in samples from different regions, batches, and extracts.

Ten milliliters of the test solution and oleanolic acid solution were mixed and dried at 80°C. The residue was reacted with 5% vanillin‐glacial acetic acid and perchloric acid at 70°C for 20 min. After cooling, ethyl acetate was added and absorbance was measured by UV spectrophotometry at 550 nm. The oleanolic acid standard curve was used to calculate total saponin content.

The total polyphenolic content of EAT and the other five extracts were determined according to the Folin‐Ciocalteu method (Eunmi et al., 2019). Two milliliters of the test solution and gallic acid standard solution were treated with 0.25 ml of 50% forinol reagent for 6 min. The solution was added to 2.5 ml of 7% Na_2_CO_3_ solution and diluted to 6 ml with water, then incubated for 1.5 hr at room temperature. The absorbance was measured by UV spectrophotometry at 760 nm. The oleanolic acid standard curve was used to calculate the total polyphenol content.

The total flavonoid content of the six extracts was measured as described by Cheng et al. (2017). Five milliliters of the test solution and the rutin reference solution were treated in turn with 0.3 ml of 5% NaNO_2_ for 5 min, 0.3 ml of 10% Al (NO_3_)_3_ solution for 6 min, 4 ml 1 M NaOH, and finally 30% ethanol was added to make up to 10 ml solution. The absorbance was measured by UV spectrophotometry at 510 nm. The rutin standard curve was used for total flavonoid content determination.

### 2.4 Ultra‐performance liquid chromatography MS analysis

Ultra‐performance liquid chromatography (UPLC) MS analysis was performed on an ion mobility‐Q‐TOF high‐resolution LC/MS instrument (Synapt G2‐Si, Waters) to study extract components. The reference standards chlorogenic acid, rutin, and quercetin were purchased from Aladdin Chemistry Co., Ltd. AT and all extracts (5 mg/ml) were dissolved in methanol, filtered through 0.45 μm, and stored at 4℃ until use. Stock solutions (500 μg/ml) of each standard and a mixture of standards (MIX‐STD, 50 μg/ml each) were prepared in 50% methanol and stored at 4℃ until use.

The analysis was performed using an LC‐20AT Prominence LC System with an LC‐20AT pump, SPD‐M20A photodiode array detector, CBM‐20A communication bus module, CTO‐20AC column oven, SIL‐20A autosampler, and YMC‐Pack ODS‐A column (4.6 × 150 mm, 5 μm) at 30℃ operating temperature. For UPLC‐MS analysis, an Acquity UPLC H‐Class system (Waters) was used with a diode array detector (DAD) (Waters) coupled with a Synapt G2 Mass Spectrometer (Q‐TOF‐MS) (Waters). The analysis was performed with a Waters BEH C18 column (100 × 2.1 mm i.d., 1.7 μm particle size) at 35℃ operating temperature at 254 nm with an injection volume of 1.0 μl. The mobile phase was water (0.1% formic acid) (Solvent A) and acetonitrile (0.1% formic acid) (Solvent B) at a 0.4 ml/min flow rate. The solvent gradient was as 5% B at 0–1.6 min, 5%‐70% B at 1.6–7.6 min, and 70%–100% B at 7.6–9.6 min, in turn. The ionization mode was ± ESI. The source temperature and desolvent gas temperature were 110℃ and 350℃, respectively. The cone gas (nitrogen) flow and desolvation gas (nitrogen) flow were 30L/h and 750 L/hr, respectively. The atomizer was 6.0 bar.

### 2.5 Anti‐inflammatory activity

Anti‐inflammatory effects of all extracts were determined using a TPA‐induced ear edema model (García‐Rodríguez et al., 2014). Male BALB/c mice (6‐week‐old, 4 per group) were provided by the Animal Experimental Center of SUN Yat‐sen University (Guangzhou, China). Mice were randomly divided into 14 groups, group‐housed (25 ± 1℃, RH 50%), and received tap water ad libitum and a standard diet. All AT extracts were dissolved in acetone and tested at concentrations of 50 mg/ml and 100 mg/ml. For the ear edema study, both mice ears were topically treated with 20 μl vehicle (acetone) or AT extract prior to treatment with 20 µl acetone or 20 µg/ml TPA in acetone. All mice were euthanized after 6 hr. Ear punches (6 mm diameter) were then harvested and weighed. All protocols were approved by the Institutional Animal Care and Use Committee (IACUC).

Ear samples were fixed in 10% formalin, decalcified in EDTA buffer, subjected to a series of dehydration cycles, and embedded in paraffin (Quan et al., 2003). Samples were cut to 4 μm and routinely treated using hematoxylin and eosin (H&E) staining. Histological changes were observed under a microscope.

The IL‐1β ELISA assay was performed using the ear edema samples. After sacrifice, mouse ear samples were homogenized over ice in phosphate buffer, then centrifuged at 8,950 *g* for 30 min. The supernatant was collected for the IL‐1β ELISA (Biosource). A capture antibody against IL‐1β was diluted with PBS then used to coat a 96‐well plate overnight at room temperature. The plate was then washed, blocked (1% BSA, 5% sucrose in PBS with 0.05% NaN_3_), and washed again. Standards were then added to the plate, leaving at least one well for background evaluation. Diluted samples (1:3–1:8) were then added to the plate and incubated for 2 hr, washed, and incubated with detection antibody for 2 hr. Streptavidin horseradish peroxidase conjugate was then added for 20 min, samples were washed, and substrate (H_2_O_2_ and tetramethylbenzidine) was added. After another 20 min incubation, stop solution (2 N H_2_SO_4_) was added and absorption was measured at 450 nm using a microplate reader.

### 2.6 Antibacterial activity

The minimal inhibitory concentration (MIC) values of EAT and the other five extracts were determined by the broth dilution method as described by Amsterdam (He et al., 2016). The McFarland standard is used for the turbidity of suspensions of micro‐organisms and bacteria for the preparation of antibacterial activity (Tian et al., 2018). The MIC values were determined against *P. aeruginosa*, *S. aureus*, *Salmonella*, *E. coli*, *B. cereus*, *White Staphylococcus*, *B. subtilis,* and *Micrococcus luteus*. The working dose of extract sample was 1,280 µg/ml, followed by 640, 320, 160, 80, 40, 20, and 10 µg/ml (w/v). An overnight culture of each strain was prepared in nutrient broth (diluted to working inoculum concentration), where 1 ml extract was added. The turbidity was compared with the McFarland values of 0.5 standards measured at 600 nm by a UV‐vis spectrophotometer. The MIC was evaluated for each extract group and is expressed in µg/ml. All tests were performed in triplicate (Gupta et al., 2015).

### 2.7 Analgesic activity

The analgesic activity of AT extracts was tested on the male Wistar rats (180–220 g) using the formalin test method (Kumar et al., 2012; Hunskaar & Hole, 1987). Rats were provided by the Animal Experimental Center of Southern Medical University (Guangzhou, China) and received tap water and a standard food diet, and were also randomized for group selection. Rats were divided into formalin, control, and AT extract groups, each with 4 mice. The blank matrix was prepared using a lipophilic cream base with a mixture of polyethylene glycol 4,000 (PEG 4,000) and liquid polyethylene glycol 400 (PEG 400) at a 2:3 ratio, with 2% (mL/g) propylene glycol as the penetration enhancer. All AT extracts were separately suspended in the blank base at 5% (w/w). Diclofenac gel (DF, 1%) was used as a reference control. The right paw of the mouse was treated topically with 0.1 g blank matrix, DF, or AT by gently rubbing with the forefinger for 1 min (Mo et al., 2013). After a further 15 min, 20 μl of 10% formalin was subcutaneously injected into the right paw. The pain response was defined as the time (in seconds) spent licking and biting the injected paw. The total licking and biting time were examined during the early phase (0–5 min) and late phase (15–30 min) after formalin injection (Sen et al., 2018).

### 2.8 Hemostatic activity

The Hemostatic activity was determined by measuring plasma recalcification time (Sui et al., 2009). All extracts were dissolved in 50% ethanol, filtered, and used at 0.5%, 1%, and 2% concentration. Blood samples were collected from New Zealand white rabbit ear‐veins, and anticoagulated with 3.8% sodium citrate (citrate: blood = 1:9, W/W). Anticoagulant solutions were centrifuged at 3,000 r/min for 30 min, then the supernatant (plasma) was collected for testing. The coagulant was 0.2775% M/40 calcium chloride solution with a plasma recalcification time within 3 min. M/40 calcium chloride solution (0.1 ml) was mixed with 0.1 ml of AT extracts sample solution in a glass tube (8 mm diameter) at 37°C. The timing was initiated when 0.1 ml of plasma was added to the coagulant‐AT solution at 37℃. The test tube was tilted once every 30 s, and the end of coagulation (recalcification time) was reached when diffuse white granular fibrin filaments appeared in the mixture solution. Each test was repeated five times.

### 2.9 Film‐form spray preparation

EAL extract (8 mg, equivalent to 18 g AT) and 5 g poly(vinyl pyrrolidone) (PVP) in anhydrous ethanol were mixed with 3 g poly (vinyl alcohol) (PVA) and purified water was added to reach a total volume of 100 ml. The solution was mixed for 30 min using a magnetic stirrer. The quality of the product was controlled by the appearance, pH value, film‐forming performance, and the content determination of active ingredients. Two grams of this product was dissolved in 15 ml purified water by sonication, and the pH value was determined. Film‐forming properties, such as film‐forming time, spray effect, film characteristics, and thickness, were applied. The main active ingredients were quantitatively determined according to the method in Section 2.3. The skin irritation was further studied. Twenty subjects, 10 males, and 10 females were selected with local skin trauma, such as scratches, abrasions, or finger barbs. Samples were sprayed on the wounds of subjects. The subjects’ responses on odor, color, and skin irritation were recorded.

## 3 RESULTS AND DISCUSSION

### 3.1 Quantitative analysis of active components

It has been reported that *A trifoliatus* (L.) Merr. contains diverse active constituents of saponins and polyphenols contributing to the beneficial biological effects, including antioxidant (Sithisarn, Muensaen, et al., 2016), anti‐inflammatory (Sithisarn, Muensaen, et al., 2016), and anticancer activities (Li et al., 2016). Among them, the flavonoids are important material basis in polyphenols for bio‐activities. Here, we established determination methods and further verified the active ingredients in different areas, batches, and extracts.

In this paper, our team tried to establish local dietary standards for this AT product. We turned to choose the simple, convenient, and feasible UV‐vis spectrophotometry for fast detection of active ingredients, which is also consistent with the national standard. The total flavonoids are determined according to Chinese Pharmacopoeia with oleanolic acid as the standard. The method of total flavonoids is mainly based on the determination method in Chinese Pharmacopoeia with the most representative flavonoid, rutin, as the standard. The total polyphenols were determined according to the Chinese national standard GB/T 8,313 with the representative gallic acid in vegetables or fruits as the standard. The methodology is validated applicable. The test solution was stable in 2 hr. As for the determination of total saponins, the standard curve was linear over the concentration range of 4–24 µg/ml with the regression equation of *y* = 0.0457*x* + 0.002 (*r* = 0.9987), where y was the absorption and *x* was the concentration. The RSD of precision validation was 1.14% (*n* = 6). The recovery was 101.68 ± 0.69% (*n* = 6). The standard curve of the total polyphenols was linear over the concentration range of 0.8333–8.3333 µg/ml examined with the regression equation of *y* = 0.0696*x* + 0.0335 (*r* = 0.9958). The recovery was 99.84 ± 1.94% (*n* = 6). The precision (RSD) was 1.81% (*n* = 6). As for total flavonoid, the standard curve was linear over the concentration range of 0.01–0.06 µg/ml examined with the regression equation of *y* = 11.7*x*–0.0217 (*r* = 0.9991). The recovery was 99.57 ± 3.26% (*n* = 6). The precision (RSD) was 0.88% (*n* = 6).

Saponins react with vanillin in the presence of perchloric acid to produce a characteristic purple‐red color.

Folin phenol reagent can oxidize the polyphenol OH group, which produces a blue‐colored solution. The flavonoids can be reduced using sodium nitrite to form a complex with aluminium nitrate, which produces a characteristic orange‐red 2‐hydroxychalone solution under alkaline conditions. The total saponin, total polyphenol, and total flavonoid contents were determined by ultraviolet spectrophotometry with ursolic acid, gallic acid, and rutin as the control substances, respectively.

**FIGURE 2:**
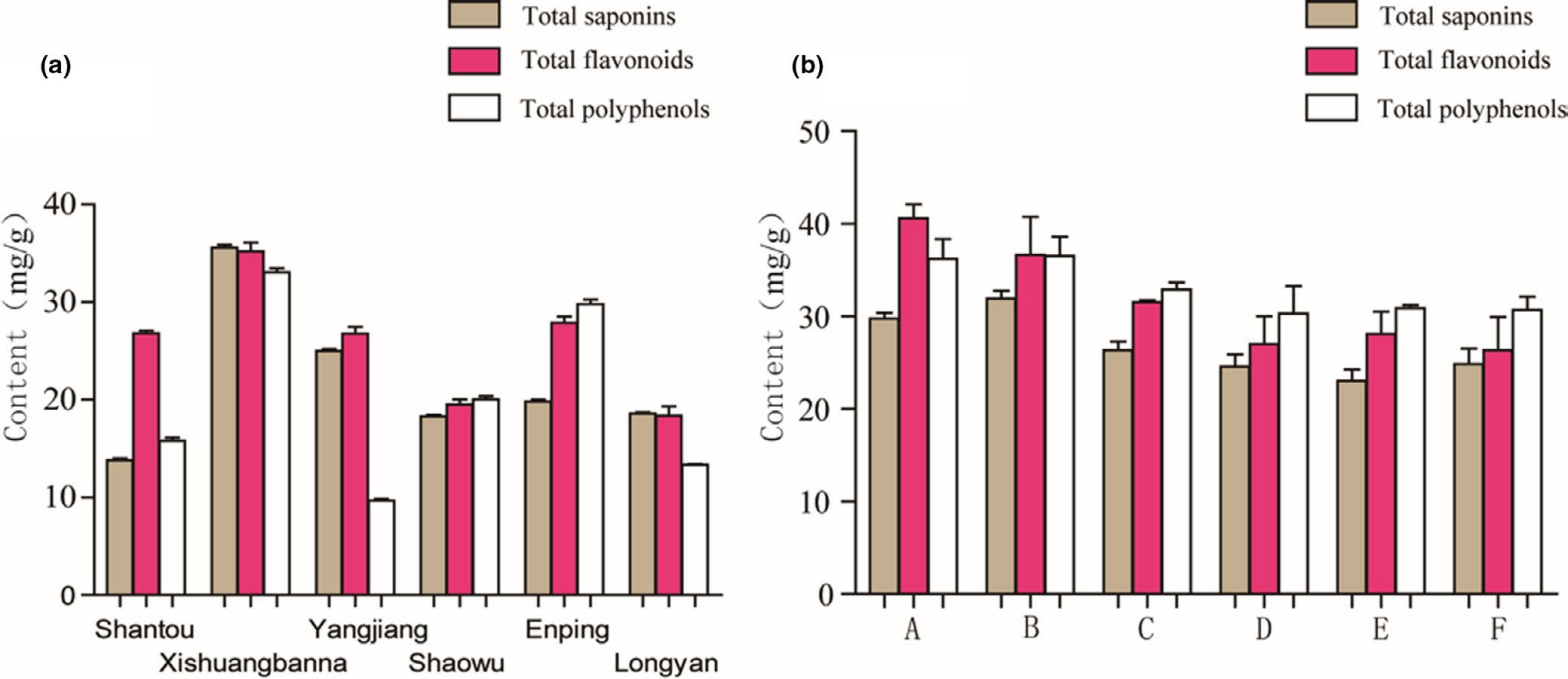
Determination of total saponins, polyphenols, and flavonoids of (a) different areas in southwest of China and (b) different batches in Enping

AT was collected from Shantou, Xishuangbanna, Yangjiang, Shaowu, Enping, and Longyan, and the total saponin, polyphenol, and flavonoid contents were determined. The content of active ingredients was different in different areas, as shown in Figure 2. The samples from Xishuangbanna and Enping contained the highest total contents of saponins, polyphenols, and flavonoids. All samples contained over 1%, 1.7%, and 1.3% of total polyphenols, total flavonoids, and total saponins, respectively. AT from Enping has been listed as a national geographical indication agricultural product with a long history of edible use and a vast area of artificial cultivation. Here, six batches of Enping AT from March to June were chosen for further analysis. Batches 20,160,315 and 20,160,401 were fresh and tender, and were collected in mid‐March and at the beginning of April. The results show that ATs were rich in saponins, polyphenols, and flavonoids, although these active ingredients decreased with the age of the vegetable. The total content of saponins, polyphenols, and flavonoids was 23–30, 30–37, and 26–41 mg/g, respectively, which indicates high nutritional value.

However, UV‐vis spectrophotometry as a rough method for the determination of total components has some shortcomings here. We will also develop more accurate methods meeting with the new national standard to improve the product quality standard.

### 3.2 HPLC and UPLC‐MS analysis of all extracts

**FIGURE 3:**
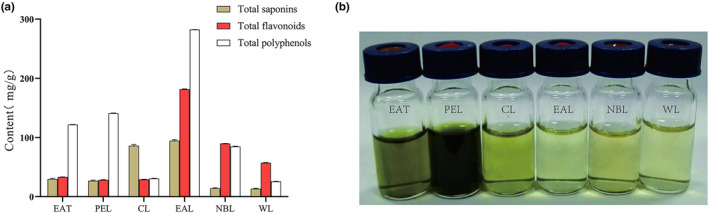
(a) Determination of total saponins, polyphenols, and flavonoids of different extracts of *Acanthopanax trifoliatus* and (b) Visual appearance of EAT, PEL, CL, EAL, NBL, and WL from the left to right

Plant extracts were crudely prepared with 95% ethanol, then further treated as shown Figure 1. The color of the solution was yellow to brown, with the PEL layer appearing the darkest (Figure 3b). The extraction rates of EAT, CL, PEL, WL, NBL, and EAL layers were 10.25%, 5.02%, 2.28%, 1.91%, 0.62%, and 0.42% of the total weight of AT powder, respectively. The extraction rate of the CL layer was the highest, and the EAL layer extraction rate was the lowest. The contents in different extracts were determined (Figure 3a). Total contents in EAT were 29.79 ± 1.08 mg/g saponins, 33.26 ± 0.17 mg/g polyphenols, and 121.85 ± 0.22 mg/g flavonoids. The contents of active ingredients in the EAL layer had the highest total saponin, total polyphenol, and total flavonoid contents of 94.57, 181.43, and 282.39 mg/g, respectively.

**FIGURE 4:**
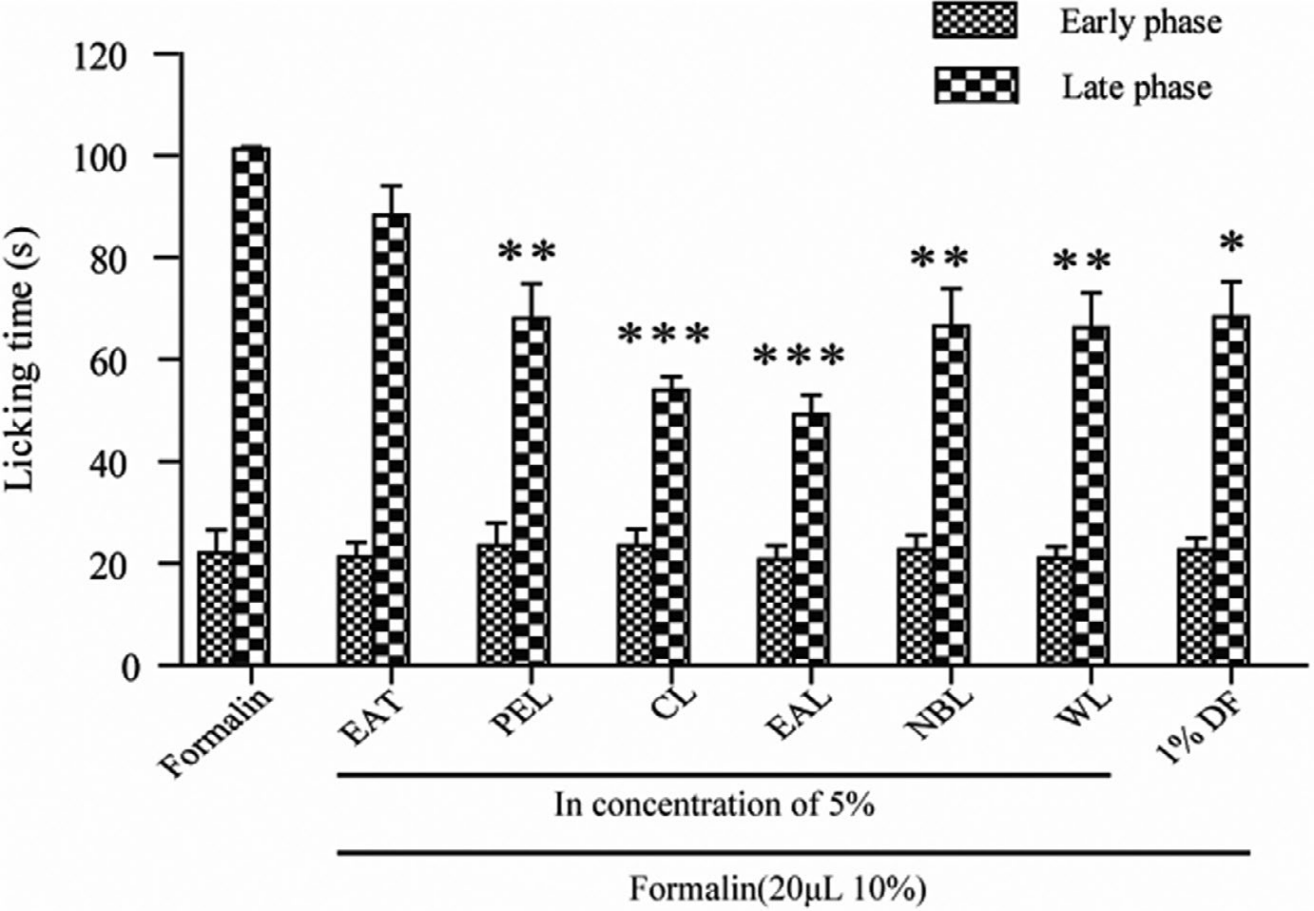
UPLC‐UV chromatograms of (a) EAT, (b) PEL, (c) CL, (d) EAL, (e) NBL, and (f) WL. Mass spectrometry analysis of the UPLC peaks confirmed the identity of the 4 peaks as (a) chlorogenic acid (molecular formula, C_16_H_18_O_9_), (b) isochlorogenic acid A (C_25_H_24_O_12_), (c) rutin (C_27_H_30_O_16_), and (d) isochlorogenic acid C (C_25_H_24_O_12_)

**FIGURE 5:**
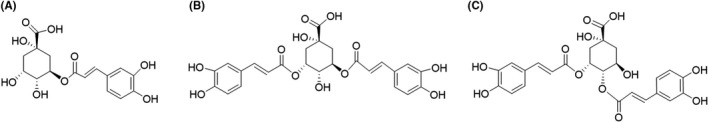
The structures of chlorogenic acid, isochlorogenic acid A and Isochlorogenic acid C, respectively

**FIGURE 6:**
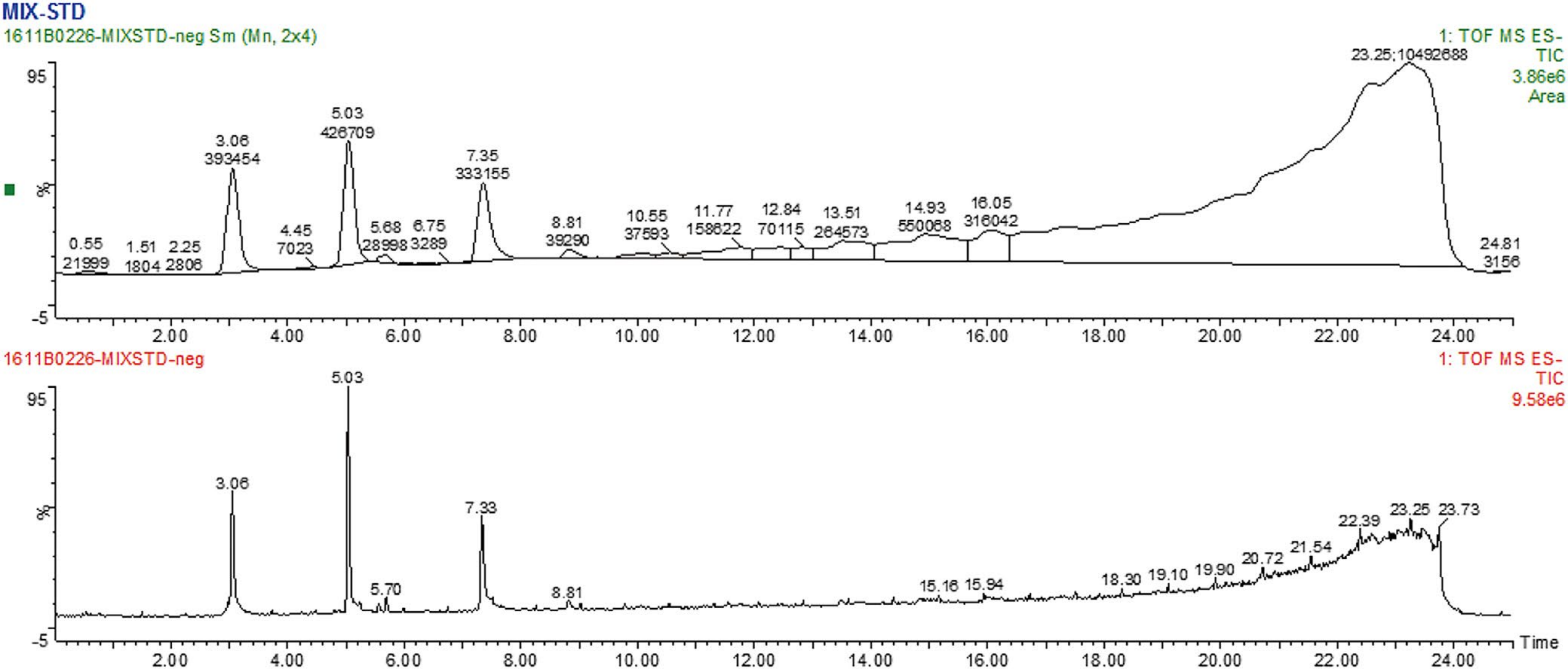
Ultra‐high liquid chromatography and mass spectrometry analysis of standard mixtures (MIX‐STD)

Acanthopanax trifoliatus contains mainly chlorogenic acid, isochlorogenic acid A, isochlorogenic acid C, rutin, and quercetin. The content of chlorogenic acid is relatively high, with 39% in fresh acanthopanax and 1. 36% in dried Acanthopanax. The chlorogenic acid and isochlorogenic acid are both important active component and widely exist in plants. One of the hydroxyl groups of chlorogenic acid esterify with caffeic acid to form isochlorogenic acid A (3,5‐dicaffeoylquinic acid) or isochlorogenic acid C (4,5‐dicaffeoylquinic acid) (Figure 4). UPLC‐MS analysis revealed the characteristic profile of various compounds that were present in AT and the different extracts (Figure 5). The constituents varied in different solvent extracts. The three peaks in MIX‐STD had the retention times (RT) of 3.06, 5.03, and 7.33 min, corresponding, respectively, to the active compounds of chlorogenic acid, rutin, and quercetin. The RT of the four main components was 3.03, 5.04, 5.61, and 5.96 min (Figure 6). The first two peaks were consistent with the peaks for the MIX‐STD, which indicates the AT extract contains chlorogenic acid and rutin, but almost no quercetin. Mass spectrometry analysis of UPLC peaks confirmed the identification of the peaks with RT of 2.99, 4.98, 5.51, 5.97, and 7.33 min as chlorogenic acid, rutin, isochlorogenic acid A, isochlorogenic acid C, and quercetin, respectively. The identity of these compounds was based on (+)‐Q‐TOF high‐resolution MS results (Table 1). Concerning chlorogenic acid, rutin, isochlorogenic acid A, and isochlorogenic acid C, the content of EAL extract was the highest in chlorogenic acid, and its analogues were significantly higher than that of other extract layers. The content of chlorogenic acid, rutin, isochlorogenic acid A, and isochlorogenic acid C in CL extract was lower than EAL extract, and the composition was more complex. PE extract contained the complex substance. The NBL and water layer contained only a small amount of rutin.

**TABLE 1:** Information for the main 4 peaks of EAT and the other five extracts tested using (+)‐Q‐TOF high‐resolution MS

Peak	UV‐RT/min	MS‐RT/min	Compound	Quasi‐molecular ion peak	Mass	Calc. Mass	Molecular formula
a	2.99	3.04	Chlorogenic acid	[M + H]^+^	355.0894	355.1029	C_16_H_18_O_9_
b	5.51	5.62	Isochlorogenic acid A	[M + H]^+^	517.1215	517.1346	C_25_H_24_O_12_
c	4.98	5.03	Rutin	[M + H]^+^	611.1518	611.1612	C_27_H_30_O_16_
d	5.97	5.98	Isochlorogenic acid C	[M + H]^+^	517.1253	517.1346	C_25_H_24_O_12_
e	7.33	7.33	Quercetin	[M + H]^+^	303.0505	303.0505	C_15_H_11_O_7_

Biological activity is closely related to a substance's active constituents. Component analysis and bioactivity experiments were therefore used to test different AT extracts. Activity experiment results showed that each extract had positive anti‐inflammatory effects. EAL extract had the strongest analgesic and hemostatic effects, and strong anti‐inflammatory activity and clear bacteriostatic effects. CL extract had the strongest anti‐inflammatory effect and strong bacteriostatic, analgesic, and hemostatic effect. PEL had the strongest bacteriostatic effect, and good anti‐inflammatory, hemostatic, and analgesic effects. NBL and WL had stronger anti‐inflammatory effects, limited bacteriostatic effects, weak analgesic effect, and no hemostatic effect. NBL and WL had low active ingredient content (except for low rutin content), had stronger anti‐inflammatory effects, limited bacteriostatic effects, general analgesic effects, and no hemostatic effect. Thus, the content of chlorogenic acid and its analogues in EAL extracts was significantly higher than in other extracts and showed stronger biological activity in terms of bacteriostatic, anti‐inflammatory, analgesic, and hemostatic effects, which is consistent with reports that suggest chlorogenic acid and its analogues have positive anti‐inflammatory effects.

The high content of active substances in AT provides a good raw material basis for product development, while ethyl acetate extract, with good bacteriostatic, anti‐inflammatory, analgesic, and hemostatic activities, has a good product development and application prospects.

### 3.3 Anti‐inflammatory activity

**FIGURE 7:**
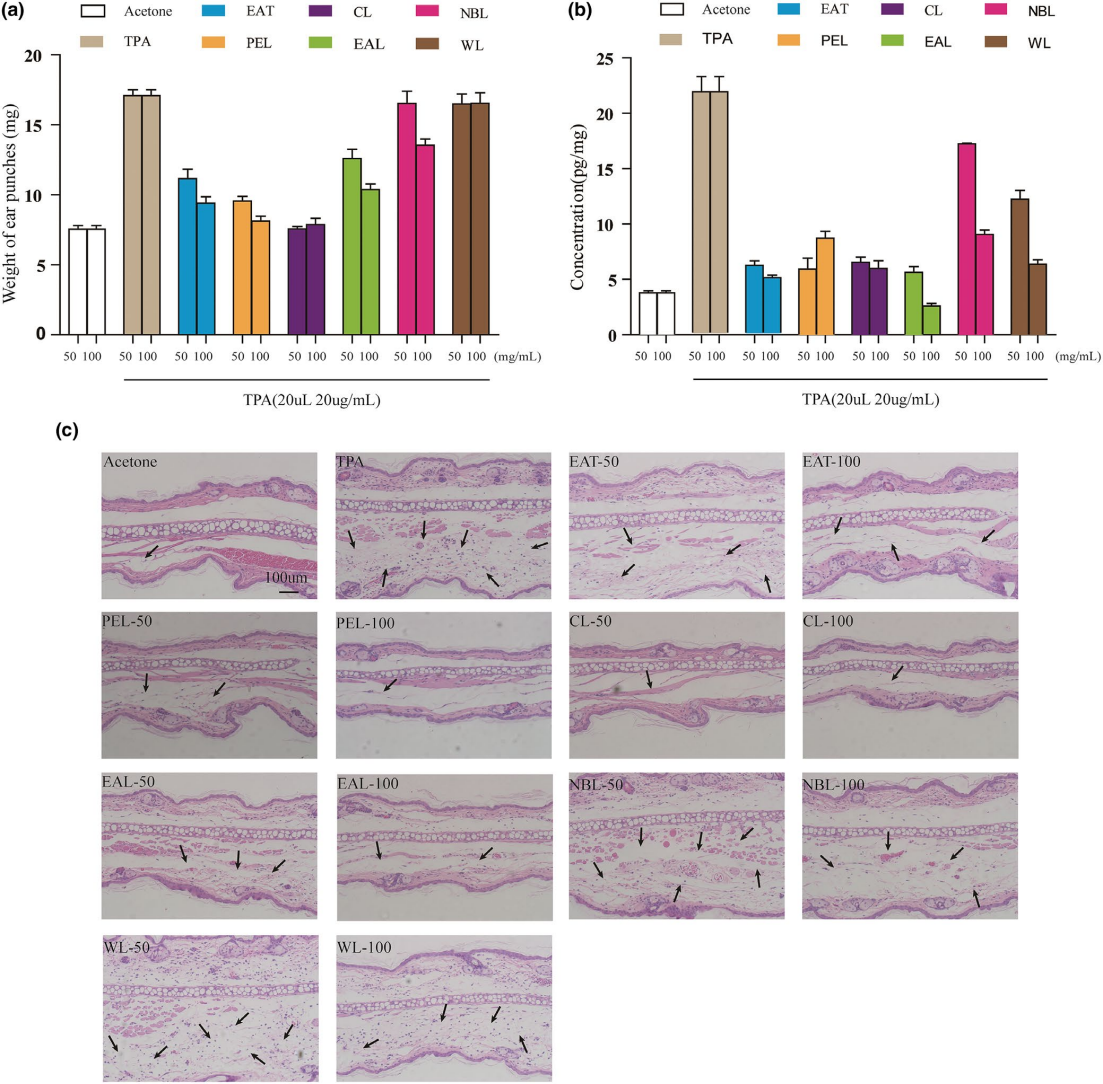
Inhibitory effect of EAT, PEL, CL, EAL, NBL, and WL on TPA‐induced ear edema in BALB/c mice. (a) The ears of BALB/c male mice were treated topically with 20 μl of acetone or AT extract (50, 100 mg/ml) dissolved in acetone, followed by topical application of 20 μl of acetone or TPA (20 µg/ml) in acetone after 6 min. All mice were sacrificed 6 hr later. Ear punches (6 mm diameter) were taken and weighed. Data are reported as mean ± *SEM* from 8 ears per group. (b) Effects of EAT, PEL, CL, EAL, NBL, and WL on IL‐1β levels. Tissue samples from the ears of mice treated with acetone, TPA, TPA + AT extract (50, 100 mg/ml) were homogenized, and the supernatants of the homogenates were analyzed using ELISA for levels of IL‐1β. Data are reported as mean ± *SEM*. (c) Effects of EAT, PEL, CL, EAL, NBL, and WL on histological changes (H&E staining) of TPA‐induced rat ear. Magnification 200×

A TPA‐induced ear edema model was used to evaluate the anti‐inflammatory activity of 50 and 100 mg/kg AT extracts. EAT, PEL, and CL significantly reduced the degree of ear swelling induced by TPA in mice at concentrations of 50 and 100 mg/kg (*p* < .001), showing positive anti‐inflammatory activity. Among all extracts, CL had the strongest anti‐inflammatory effect, and its inhibition rate reached almost 99.9%, even at concentrations of 50 mg/kg. EAL also significantly reduced TPA‐induced ear swelling in mice at 50 and 100 mg/kg, with inhibition rates of 47.1% and 71.9%, respectively. In addition to CL and WL, EAT, PEL, EAL, and NBL at a dose of 100 mg/kg have a higher ability to reduce ear swelling than at a dose of 50 mg/kg. NBL had its strongest activity only at 100 mg/kg concentration, while WL and NBL at 50 mg/kg had almost no anti‐inflammatory effect. We next determined the histopathologic changes in the mouse ear by paraffin section and HE staining. As was shown in (Figure 7c), extensive edema and severe inflammatory cell infiltration were observed in the TPA group. Treatment with CL, EAL, and PEL led to an obvious decrease in the degree of ear swelling and infiltrating cells at concentrations of 50 and 100 mg/ml.

TPA can induce keratinocytes in mouse ear tissue to activate serine/threonine protein kinase and NF‐κB, producing inflammatory factors such as TNF‐α, IL‐1β, IL‐2, and IL‐6 (Du et al, 2013). In addition, inflammatory factors can promote secondary inflammatory cytokine production and aggravate the inflammatory response. Based on the possible mechanism behind TPA inducing inflammation, and on previous studies, the level of IL‐1β was investigated by ELISA in TPA‐induced mice ears (Figure 7a,b). ELISA results from pro‐inflammatory markers in ear homogenates revealed that TPA exposure significantly increased interleukin‐1β (IL‐1β) levels. Compared with the TPA and the blank acetone group, all AT extracts effectively lowered IL‐1β protein expression (*p* < .001) at 50 and 100 mg/kg. ELISA results confirmed the positive anti‐inflammatory activity of AT, supporting its important role in the prevention and treatment of tumors and other diseases.

### 3.4 Antibacterial activity

Antimicrobial activity was studied on *P. aeruginosa*, *S. aureus*, *salmonella*, *E. coli*, *B. cereus*, *White Staphylococcus*, *B. subtilis*, and *Micrococcus luteus*. NBL and WL extracts had no obvious inhibitory effect. PEL extracts exhibited a strong inhibitory effect on all the bacteria with a MIC range of 10 to 80 µg/ml, and the strongest inhibitory effect on *Salmonella,* close to the effect of ciprofloxacin. Therefore, extracts could be further developed to study new antimicrobial agents. The inhibitory effect of CL extract on *Salmonella* and *E. coli* was slightly lower than that of PEL extract. EAL extract has bacteriostatic activity, with stronger effects on *Salmonella* and *E. coli* than CL (Table 2). Among them, the PEL has the best antibacterial effect. The PEL has a strong inhibitory effect on *Salmonella*, *Escherichia coli*, *Staphylococcus aureus*, *Staphylococcus albicans*, and *Micrococcus luteus*. Its minimum inhibitory concentration (MIC) is 10 μg/ml, but its inhibitory effect on Bacillus is poor.

**TABLE 2:** MICs of AT extracts for Gram‐positive and Gram‐negative bacteria

Species	MIC (µg/mL) of						
	EAT	PEL	CL	EAL	NBL	WL	Ciprofloxacin
*P. aeruginosa*	1,280	80	160	640	640	>1,280	<10
*S. aureus*	640	40	160	320	640	1,280	<10
*Salmonella*	640	10	20	160	640	>1,280	<10
*E. coli*	640	20	40	160	1,280	1,280	<10
*Bacillus cereus*	1,280	80	160	320	1,280	>1,280	<10
*White staphylococcus*	640	20	80	320	640	1,280	<10
*Bacillus subtilis*	>1,280	80	320	1,280	>1,280	>1,280	<10
*Micrococcus luteus*	>1,280	40	160	640	>1,280	>1,280	<10

### 3.5 Analgesic activity

**FIGURE 8:**
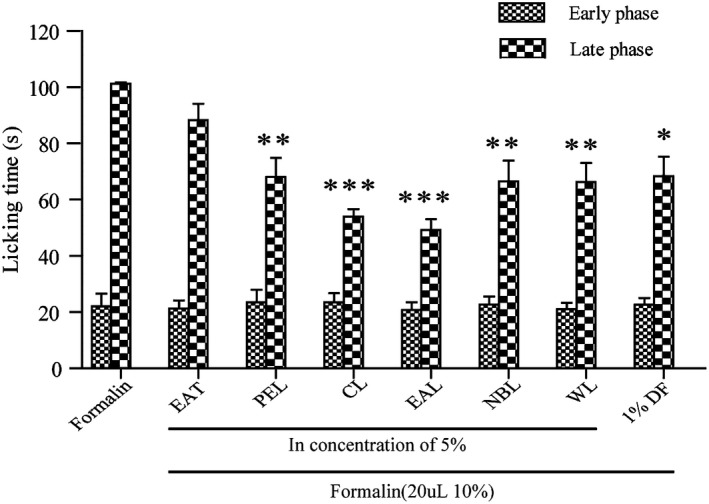
Analgesic effects of topical formulations of different extracts in the formalin test. Data are reported as mean ± *SEM* (*n* = 5). Differences between formalin and extract treated groups were analyzed using a Student–Newman–Keuls one‐way ANOVA (*p < .001; **p < .01; ***p < .001)

Analgesic effects were observed by measuring behaviors demonstrating pain relief in formalin‐induced rats with 1% diclofenac sodium gel (DF) as the positive control substance. Mouse foot licking was measured during 0–5 min (first stage) and 15–30 min (second stage) after formalin injection. The more the mice licked their feet, the more pain they experienced. The results showed that all groups, including the DF group, did not effectively show reduced pain during the first stage, with licking times at approximately 22 s. As shown in Figure 8, the groups that received 5% PEL, CL, EAL, NBL, WL, and DF displayed a significantly lower licking time during the late phase. The CL and EAL groups exhibited stronger analgesic efficacy than the positive control group. The analgesic effect of EAL was the strongest. In the second stage, the pain inhibition rate reached 51.4%. The effects of 5% PEL, NBL, and WL were similar to 1% DF during the second stage. Except for EAT, the different extracts of AT in the second stage had statistical differences compared with the control group (*p* < .01, *p* < .001), and the mean values were lower than those of the control group. Therefore, the experimental results demonstrate that AT extracts have good analgesic activity.

### 3.6 Hemostatic activity

**FIGURE 9:**
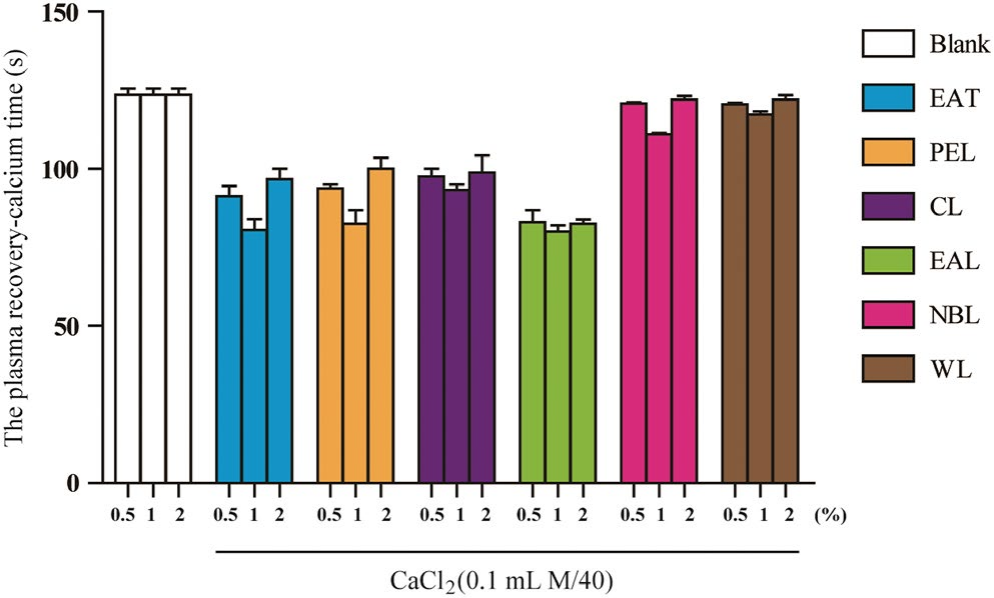
Determination of plasma recovery‐calcium time. Data are reported as mean ± *SEM* (*n* = 5)

AT hemostatic effects were evaluated at concentrations of 0.5%, 1.0%, and 2.0% by the plasma recalcification time (CT) of arterial blood from New Zealand white rabbits (Figure 9). Under these conditions, the shorter the plasma recalcification time was, the stronger the hemostatic activity. All extracts had stronger hemostatic activity at a concentration of 1% compared with 0.5% and 2%. The plasma recalcification time varied between groups at 1.0% concentration. Among these, EAL extract exhibited the shortest plasma recalcification time of approximately 80 s, which was 34.85% less than the blank group. The plasma recalcification time was also significantly shortened in the crude ethanol extract, PEL extract, CL extract, and EAL extract, while the effect of NBL extract and WL extract had no obvious effects. We have proved for the first time that the EAL extract of AT has a good hemostatic effect, and it is very promising to develop AT for hemostatic function in the future.

### 3.7 Film‐form spray preparation

The ethanol crude extract of AT further refined by ethyl acetate showed the best analgesic and hemostatic activity according to our research, which is significant for wound treatment. Spray film agent is a kind of commonly used dosage form for trauma treatment. We tried to develop a stable and controllable film spraying agent of AT for wound treatment.

Our spraying agent has the characteristics of simple preparation process, convenient carrying, and transportation. Orthogonal design was used in our previous study (H. Lin & H. Hu, unpublished) to optimize PVA, PVPK30, and ethanol dosages with the evaluation indexes of film‐forming time, spraying effect, film state, and film thickness. The optimum blank matrix prescription is 3% PVA, 5% PVP, and 35% ethanol. The optimized blank matrix is stable and transparent, which is easy to spray and has film‐forming properties for EAL‐loading. The film‐forming EAL extract spray is a light green translucent solution at pH 5–7. The film‐forming time of self‐prepared samples should be within 15 min at 37℃. The film is uniform, dispersed, smooth with a predictable thickness. Three batches of spray solution were prepared and the content of total flavonoids (i.e., 20.59%, 20.86%, and 20.10%) was determined for use as the main quality control standard. Thus, the prepared spray formed a dense film around the wound, so as to prevent the entry of external bacteria and dust, and maintain a moist and healthy environment around the wound.

A skin irritation test on human subjects without a wound was carried out, and the results were satisfactory (Table 3). The skin irritation test examined the volunteers' perception of odor, color, and skin irritation. According to the feedback, the product had a slight alcoholic taste. The color of the product was light and nearly colorless. One of the subjects felt slight irritation, which may be due to individual skin discomfort caused by ethanol in the product.

**TABLE 3:** Subject evaluations of this product

Gender	Odor	Color	Irritation
Male	Ten male volunteers thought the product had a slight alcoholic taste.	Ten volunteers considered the product was colorless to light green.	Ten volunteers thought the product was not irritating.
Female	Nine volunteers thought the product had a slight alcoholic taste, one volunteer thought it was tasteless.	Ten volunteers considered the product was colorless to light green.	Nine volunteers thought it was not irritating, and one volunteer thought it was irritating.

Thus, the prepared spray agent has analgesic and hemostatic effects. And it is portable and fast, and offers good permeable and continuous wound care.

## 4 CONCLUSIONS

In this study, the active ingredients in AT were analyzed and biological activities were further explored. PEL produced the most effective antibacterial activity with a lower MIC than that of other extracts. EAL had excellent hemostatic and strong analgesic activities. CL had the strongest anti‐inflammatory effects and could significantly reduce the expression of IL‐1β protein in mouse ear tissue. EAL and CL extracts produced analgesic effects during the late phase, which were stronger than that of DF. Above all, the ethyl acetate layer extract had the highest content of active substances and displayed greater anti‐bacterial, anti‐inflammatory, analgesic, and hemostatic effects, among which analgesia and hemostasis were the strongest. Based on the component analysis of different extracts, the relationships between the substance components and pharmacodynamics were analyzed. These results provide a reliable theoretical and practical basis for the research and development of the topical application of AT products.

## CONFLICT OF INTEREST

The authors declare that they have no conflict of interest.

## ETHICAL APPROVAL

Animal procedures experiments were approved by the Animal Care and Use Committee of Guangzhou University of Technology.
